# Utilization of CRISPR-Mediated Tools for Studying Functional Genomics in Hematological Malignancies: An Overview on the Current Perspectives, Challenges, and Clinical Implications

**DOI:** 10.3389/fgene.2021.767298

**Published:** 2022-01-28

**Authors:** Maheswaran Solayappan, Adam Azlan, Kang Zi Khor, Mot Yee Yik, Matiullah Khan, Narazah Mohd Yusoff, Emmanuel Jairaj Moses

**Affiliations:** ^1^ Regenerative Medicine Sciences Cluster, Advanced Medical and Dental Institute, Universiti Sains Malaysia, Penang, Malaysia; ^2^ Department of Biotechnology, Faculty of Applied Sciences, AIMST University, Bedong, Malaysia; ^3^ Department of Pathology, Faculty of Medicine, AIMST University, Bedong, Malaysia

**Keywords:** clustered regularly interspaced short palindromic repeats (CRISPR), gene editing, hematological malignancies, functional genomics, therapeutic targets

## Abstract

Hematological malignancies (HM) are a group of neoplastic diseases that are usually heterogenous in nature due to the complex underlying genetic aberrations in which collaborating mutations enable cells to evade checkpoints that normally safeguard it against DNA damage and other disruptions of healthy cell growth. Research regarding chromosomal structural rearrangements and alterations, gene mutations, and functionality are currently being carried out to understand the genomics of these abnormalities. It is also becoming more evident that cross talk between the functional changes in transcription and proteins gives the characteristics of the disease although specific mutations may induce unique phenotypes. Functional genomics is vital in this aspect as it measures the complete genetic change in cancerous cells and seeks to integrate the dynamic changes in these networks to elucidate various cancer phenotypes. The advent of CRISPR technology has indeed provided a superfluity of benefits to mankind, as this versatile technology enables DNA editing in the genome. The CRISPR-Cas9 system is a precise genome editing tool, and it has revolutionized methodologies in the field of hematology. Currently, there are various CRISPR systems that are used to perform robust site-specific gene editing to study HM. Furthermore, experimental approaches that are based on CRISPR technology have created promising tools for developing effective hematological therapeutics. Therefore, this review will focus on diverse applications of CRISPR-based gene-editing tools in HM and its potential future trajectory. Collectively, this review will demonstrate the key roles of different CRISPR systems that are being used in HM, and the literature will be a representation of a critical step toward further understanding the biology of HM and the development of potential therapeutic approaches.

## Introduction

Hematological malignancies (HM) are cancers that begin in the cells of blood-forming tissues such as the bone marrow or generally in the cells immune system. In the revised World Health Organization (WHO) classification, hematological malignancies are divided according to morphology, immunophenotype, and genetic and clinical features (Taylor et al., 2017). Further divisions can be made which are myeloid malignancies that are categorized into the following groups: myeloproliferative neoplasms, myelodysplastic/myeloproliferative neoplasms, myelodysplastic syndromes, acute myeloid leukemias, acute leukemias of ambiguous lineage, and precursor lymphoid neoplasms. Then there are lymphoid malignancies that are categorized into mature B-cell neoplasms, mature T- and NK-cell neoplasms, Hodgkin’s lymphoma, histiocytic and dendritic cell neoplasms, and posttransplantation lymphoproliferative disorders.

The current advancement in the field of molecular biology has spurred the way toward understanding a disease in an extensive manner and especially deepens our knowledge of hematological malignancies. Many advanced molecular biology and bioinformatics tools such as PCR, NGS, and karyotyping are currently utilized to understand the pathogenesis of hematological malignancies. Nevertheless, these methods only allow us to map genes but not fully understand the function. Therefore, it is imperative for researchers to utilize functional genomics to decipher and unravel new mechanisms that were previously unknown.

Advancement in molecular biology has now paved the way for us to understand the roles of genes that are involved directly or indirectly in the molecular and cellular mechanisms underlying hematological malignancies. The way forward now is to take the optimal charge through unraveling the roles of specific genes by manipulating their functions through gene editing. Currently, there are many techniques available to study gene function including site-directed mutagenesis and siRNA-mediated gene silencing (Gavrilov and Saltzman, 2012).

Nevertheless, these techniques are regarded to be less efficient and specific as compared to the various gene editing techniques (Zhang and McCarty, 2017) which will be reviewed in the next section.

## Gene Editing Techniques

Gene editing is the ability to make extremely precise changes in the DNA sequence of a living organism and essentially customizing its genetic makeup. Gene editing is achieved by using enzymes, predominantly nucleases, that have been synthesized to target a specific DNA sequence. This is when they introduce cuts into the DNA strands, allowing the removal of existing DNA and the insertion of a replacement DNA. It is a type of genetic engineering tool to insert, delete, or replace a DNA in the genome of an organism using “molecular scissors” (Saha et al., 2019). These nucleases create site-specific double-strand breaks (DSBs) at desired locations in the genome (Vítor et al., 2020). The induced double-strand breaks are then repaired through non-homologous end-joining (NHEJ) or homologous recombination (HR) which results in targeted mutations also known as “edits” (Salsman et al., 2017). There have been three important families of bio-engineered nucleases being used such as zinc-finger nucleases (ZFNs), transcription activator-like effector-based nucleases (TALENs), and CRISPR-Cas systems (Li et al., 2020).

### Zinc-Finger Nucleases

The discovery of zinc-finger nucleases (ZFN) in the 1980s added value to the gene editing approach by being a precision tool in genome editing: it carried a site-specific recognition pattern in editing the desired gene. The ZFNs are composed of two parts, namely, Fokl nuclease that is fused with zinc-finger DNA-binding domains. The zinc-finger DNA-binding domain has a unique characteristic of recognizing a 3-base pair site on DNA, and it can be combined to recognize longer sequences (Wu et al., 2007). Furthermore, the ZFNs act as dimers by upsurging the length of the DNA recognition site and increasing the specificity. Cys_2_-His_2_ ZFNs are fusions between a custom-designed Cys_2_-His_2_ zinc-finger protein and the cleavage domain of the FokI restriction endonuclease. Cys_2_-His_2_ ZFPs bind DNA by inserting an *α*-helix into the major groove of the double helix (Wu et al., 2007) ZFNs function as dimers, with each monomer recognizing a specific “half site” sequence, typically nine to 18 base pairs of DNA, *via* the zinc-finger DNA-binding domain. One major concern associated with the use of ZFNs for genome editing is off-target mutations (Chen et al., 2014). As a result, several approaches have been undertaken to enhance their specificity; among the most successful of these has been the creation of obligate heterodimeric ZFN architectures that rely on charge–charge repulsion to prevent unwanted homodimerization of the FokI cleavage domain, thereby minimizing the potential for ZFNs to dimerize at off-target sites. Additionally, protein-engineering methods have been used to enhance the cleavage efficiency of the FokI cleavage domain (Chen et al., 2014). The main hurdle in using ZFNs was the 3-base pair site on DNA requirement that made the design more challenging (Gupta and Musunuru, 2014). The upper hand was the guanine-rich target sites that appeared to be more efficient at editing when compared to the non–guanine-rich sites. Furthermore, the ZFN interaction with DNA is modular, and each ZF interacts with DNA independently that hampered the editing efficiency. Therefore, scientists needed to address these issues if they wanted to have more efficiently edited genome. According to a study which adopted the ZFN approach for genome editing in human pluripotent stem cells, the scientists observed multiple off-target genomic sites based on high-sequence similarity to the on-target site and found a single off-target mutation in the 184 clones assessed (Hockemeyer et al., 2009). Therefore, scientists should be aware of the likelihood that ZFNs that are designed for a purpose may experience undesired off-target effects at a low rate. There are ways to reduce off-target effects, by using a pair of ZFNs that have different FokI domains that are obligate heterodimers. Through this approach, we will be able to prevent a single ZFN from binding to two adjacent off-target sites and in turn generating a DSB. The second approach would be the introduction of purified ZFN proteins into cells. Although it is efficient at inducing DNA edits, ZFNs are cumbersome and laborious to assemble. Currently, the ZFN approach is still at the early stage and there are some difficulties that need to be addressed and sorted off before it is clinically used to treat human diseases. ZFN-based strategies for gene editing of human cells may provide a viable option to treat human disease in the future.

### TALENs (TALE Nucleases)

Transcription activator-like effector nuclease (TALEN) has rapidly emerged as an alternative to ZFNs for genome editing, and they are bacterial effector proteins. In 2009, the code used by TALE proteins to recognize DNA was uncovered. TALE DNA–binding domains can be constructed using a variety of methods, with the most straightforward approach being the Golden Gate assembly (Maeder et al., 2013). TALENs comprise a non-specific DNA cleavage domain fused to a customizable sequence-specific DNA-binding domain to generate DSBs. This DNA-binding domain consists of a highly conserved repeat sequence from transcription activator-like effector (TALE), which is a protein originally discovered in the phytopathogenic *Xanthomonas* bacteria that naturally alters the transcription of genes in host plant cells. The binding of TALE to DNA is mediated by a central region that contains an array of 33 to 35 amino acid sequence motifs. The amino acid sequence of each repeat is structurally similar, except for two hypervariable amino acids (the repeat variable di-residues or RVDs) at positions 12 and 13. DNA-binding specificity is determined by RVDs, with ND specifically binding to C nucleotides, HN to A or G nucleotides, NH to G nucleotides, and NP to all nucleotides. There is a one-to-one correspondence between RVDs and contiguous nucleotides in the target site, constituting a strikingly simple TALE–DNA recognition cipher.

Functional endonuclease FokI is factitiously fused to DNA-binding domains to create site-specific DSBs and thereby stimulate DNA recombination to achieve TALEN-induced targeted genomic modification. To cleave the two strands of the targeted DNA, the FokI cleavage domain must be dimerized. Hence, like zinc fingers, such a TALEN module is designed in pairs to bind opposing DNA target loci, with proper spacing (12–30 bp) between the two binding sites. However, compared to zinc-finger proteins, there is no need to redesign the linkage between repeats constituting long arrays of TALEs, whose function is to target individual genomic sites. Following pioneering studies on zinc-finger proteins, multiple effector domains have become accessible to support the fusion of TALE repeats for different genomic modification purposes, including nucleases, transcriptional activators, and site-specific recombinases. Although their simpler cipher codes provide better simplicity in design than triplet-confined zinc-finger proteins, one of the primary technical hurdles for cloning repeat TALE arrays is the design of identical repeat sequences on a large scale. To address this limitation, a few strategies have been established to facilitate the fast assembly of custom TALE arrays, including “Golden Gate” molecular cloning, high-throughput solid phase assembly, and connection-independent cloning techniques. More recent advances in TALEN assembly, though, have focused on the development of methods that can enhance their performance, including specificity profiling to uncover non-conventional RVDs that improve TALEN activity, directed evolution as means to refine TALE specificity, and even fusing TALE domains to homing endonuclease variants to generate chimeric nucleases with extended targeting specificity (Liu et al., 2014). TALENs attach FokI to arrays of DNA-binding modules, originally from plant pathogens, that each targets a single base pair. TALENs are smaller than Cas9 but larger than ZFNs. The modules have high DNA-binding affinity but include repeated sequences that create cloning challenges. According to a study conducted by Bethany K. R. and Randall S. P. in 2015, they have stated that TALENs are not efficient at making biallelic modifications, and it requires further cloning steps or alternative animal breeding step to produce animals with the intended biallelic mutations.

### CRISPR

Although recently developed programmable editing tools such as zinc-finger nucleases and transcription activator-like effector nucleases have significantly improved the capacity for precise genome modification, these techniques have limitations. The new kid on the block, CRISPR (clustered regularly interspaced short palindromic repeats) technology represents a significant improvement over these, reaching a new level of targeting, efficiency, and ease of use. CRISPR nucleases lead the gene-editing platform because they are the most powerful and direct to use gene-editing tools available now. Based on their *cas* gene content, CRISPR-Cas systems can be classified into six major types (I–VI) (Koonin et al., 2017). The systems are classified into two general parts, namely, clustered regularly interspaced short palindromic repeats (CRISPR) arrays and CRISPR-associated (Cas) proteins. The CRISPR immunological signature represents the memory of the previous infections encoded within individual spacers separated by conserved repeats. Cas proteins carry out the adaptive immune functions and are highly diverse, resulting in CRISPR-Cas systems currently being grouped into two classes, six types, and over 30 subtypes (Wimmer and Beisel, 2020). The speedy unearthing and continuous development has led to the availability of choices; hence, choosing the right nuclease for an experiment is a mandatory part of this approach. The CRISPR system allows for site-specific genomic targeting in virtually any organism (Guilinger et al., 2014). It also offers platform that is an efficient way of making precise genetic changes to the human genome. This can be employed for disruption, addition, and correction of genes, thereby enabling a new class of genetic therapies that can be applied to hematological disorders.

#### Cas 9 System

The CRISPR-Cas9 system, which has a role in adaptive immunity in bacteria, is the most recent addition to the genome-editing toolbox. The primal CRISPR gene editor is from *S. pyogenes* Cas9, and it still remains to be the most popular CRISPR nuclease for the gene editing approach. The SpCas9 is a colossal CRISPR nuclease bearing 1,368 amino acids in size and possesses a moderately relaxed protospacer adjacent motif (PAM; 5′-NGG) as compared to its orthologs. SpCas9 happens to be the most well-characterized of all CRISPR systems due to it being the first to be repurposed as a gene editor. Thus, making it readily available in virtually any format (plasmid, mRNA, and protein) and in-line with other genome engineering applications. In bacteria, the type-II CRISPR system provides protection against DNA from invading viruses and plasmids *via* RNA-guided DNA cleavage by Cas proteins (Guilinger et al., 2014). Short segments of foreign DNA are integrated within the CRISPR locus and transcribed into CRISPR RNA (crRNA), which then anneal to *trans*-activating crRNA (tracrRNA) to direct sequence-specific degradation of pathogenic DNA by the Cas9 protein. This system has since been simplified for genome engineering and now consists of only the Cas9 nuclease and a single guide RNA (gRNA) that contains the essential crRNA and tracrRNA elements (Maeder et al., 2013).

There are many naturally-occuring Cas9 ortologs available for gene-editing purposes. There are three types of SpCas9, namely, SpCas9 with relaxed PAM (SpCas9-NG), SpCas9 nickase (Cas9n), and nuclease-dead SpCas9 (dCas9). In order to differentiate these ortologs from SpCas9, there are two main characteristics which are size and PAM specificity. The size really matters for hard-to-transfect cell lines which are vectors that easily exceed 10 kb as well as AAV applications where AAV can only package ∼4.5 kb. Compact Cas9 ortologs include those isolated from *S. aureus* (SaCas9) and *C. jejuni* (CjCas9), both of which can be packaged in AAV. SpCas9-NG is engineered with valine to disrupt the interaction between Cas9 and the second G of the 5′-NGG PAM. There is additional modification done to this SpCas9-NG based on molecular modeling to restore the cleavage activity of this “relaxed” Cas9 variant (Nishimasu et al., 2018). The second type, SpCas9 nickase, is engineered with a single alanine substitution that transforms Cas9 into a “nickase” that can only cut one strand of target DNA. In order to create a double-stranded break in gene editing, a pair of Cas9 nickase is used as it gives fewer off-target DSBs than SpCas9 (Tsai and Joung, 2016). The dCas9 has two alanine substitutions to eliminate Cas9’s nuclease activity but its DNA targeting ability is not affected. This synthetic construct that is generated by tethering transcriptional activation domains to a dCas9 can be directed to the promoters of endogenous target genes by single guide RNAs (sgRNAs) to activate transcription. It can also be used to target various effector domains can be fused to dCas9 to enable programmable transcriptional and epigenetic control (Li et al., 2019).

PAM specificity, on the other hand, dictates the available ‘target space’ for each ortolog or how many guide RNA (gRNA) binding sites there are in a given genome for a given PAM. Ortologs with simple PAMs can interact with more genomics sites compared to those with stringent PAMs. For example, there are more instances of 5′-NGG in any given genome as opposed to 5′-NGGNG which is the PAM site for St3Cas9 (*Streptococcus thermophilus*). Relaxed PAMs are good for more flexibility in the gRNA design, but it should be avoided because it may lead to off-target activity. When the condition allows for increased gRNA-binding sites, it will lead to higher potential off-target activity. However, like any emerging technology, the CRISPR-based assay system has minor potential pitfalls, including promiscuous off-target activity by Cas9. To address this issue, various preventative strategies have been employed, such as introducing purified Cas9 straight into target cells, using Cas9 nickase (Cas9n), decreasing sgRNA sequences by 2–3 nt, and exploiting additional two guanines at the 5ˊ terminus of gRNA immediately juxtaposed to the target-complementary region (Concordet and Giovannangeli, 2017).

#### Cas10 System

Classification of CRISPR-Cas10 variants that would capture their evolutionary relationships to the maximum possible extent is essential for comparative genomic and functional characterization of this theoretically and practically important system of adaptive immunity (Chen et al., 2014). The Cas10 enzyme is classified into Class 1 CRISPR-Cas systems which consist of the three types, type I, III, and IV systems as well as 22 subtypes. Along with Cas10 in Class 1, it also includes Cas3 and Cas8-like (csf1) enzyme which are considered signature genes in types I, III, and IV, respectively (Vangah et al., 2020). Type III systems, which are identified by their signature Cas10 gene, are further divided into subtypes: III-A/D, which encodes the Cas10-Csm complex, and III-B/C, which encodes the Cas10-Cmr complex. Type III effector complexes employ a uniquely elaborate targeting mechanism (Pyenson et al., 2017; Tamulaitis et al., 2017) in which active transcription of the target sequence is required for CRISPR immunity (Deng et al., 2013). The large subunit of type III systems, Cas10, is a protein containing two RNA recognition motif domains, whereby one domain shows a high significant similarity to the palm domain, the catalytic domain of a broad variety of RNA and DNA polymerases and nucleotide cyclases. This palm domain is predicted to be an active enzyme, whereas the second RRM domain is inactivated form (Koonin et al., 2017).

#### Cas13 System

DNA targeting CRISPR enzymes, such as Cas9 and Cas12a, have enabled many new possibilities for manipulating and studying DNA. Recent computational efforts to identify new CRISPR systems uncovered a novel type of RNA targeting enzyme, Cas13. The diverse Cas13 family contains at least four known subtypes, including Cas13a, Cas13b, Cas13c, and Cas13d (Elliott et al., 2021). One of the most straightforward applications of Cas13 *in vivo* is targeted RNA knockdown using mammalian codon optimized Cas13 and guide expression vectors. Knockdown of RNA relies on cleavage of the targeted transcripts by the endogenous RNase activity of the dual HEPN domains of the protein, the efficiency of which varies between different orthologs and subtypes of Cas13. The Cas13a is an RNA-guided, single-component enzyme that possesses two higher eukaryotes and prokaryotes nucleotide-binding (HEPN) domains that target single-stranded RNA (ssRNA). It encompasses a functionally distinct nuclease that is responsible for catalyzing crRNA maturation to form a Cas13a:crRNA complex that is competent for target RNA binding. The binding of the said complex to a complementary ssRNA is termed as activator-RNA. This binding activates the HEPN-nuclease for both target and general ssRNase activity. The action of the HEPN-nuclease is repressed by fractional occlusion of the HEPN active site until binding to the activator-RNA occurs. This effectively makes the activator-RNA the allosteric switch for RNase activity (Narimani et al., 2019). While the mechanism appears conserved between homologs, Cas13a enzymes can be functionally separated into two distinct subtypes based on their processing activity and HEPN-nuclease nucleotide preference (Huang et al., 2018). As a result, guide design and restrictions on targeting depend on the system used (Abudayyeh et al., 2017). In a study conducted by Cox et al. (2017), they expanded the RNA targeting ability of Cas13 to direct ADAR2 deaminase for RNA base (adenosine to inosine) modifications in human cells to recover functional proteins and halt disease progression. The application of Cas13a for nucleic acid detection and targeting are active Cas13a HEPN nuclease, which will turn over multiple ssRNA substrates, a biochemical behavior that can be leveraged for signal amplification in target detection by coupling Cas13a activation to specific ssRNA reporter cleavage, resulting in liberation of a quenched fluorophore. Expanding on this, the specific high-sensitivity enzymatic reporter unlocking (SHERLOCK) platform was developed as a tool for nucleic acid detection (Narimani et al., 2019). The method starts with RNA sequence amplification *via* RPA or reverse transcriptase RPA (rt-RPA), before incubating the sample with Cas13a and reporter probes, and fluorescence is then measured. SHERLOCK can detect nucleic acids in patient biofluids down to low atto molar concentrations, allowing the detection of tumor mutation in cell-free DNA (cfDNA). In mock cfDNA samples, SHERLOCK can detect two cancer mutations, EGFR L858R and BRAF V600E, under low allelic fraction with single-base mismatch sensitivity. In addition to *in vitro* RNA target detection, catalytically inactive Cas13a retains its RNA-binding activity such that it can be coupled to a fluorescent probe to enable live cell RNA tracking. This provides an alternative method to recognize and visualize RNA (Pandolfi, 2001).

#### CRISPRainbow System

In the past, there was a vacuum in studying and imaging multiple genomic loci in living cells at a time. This created a limitation for us in exploring the biological chromodynamics or chromosome dynamic. CRISPRainbow is a system for labeling DNA in human cells based on nuclease-dead (d) Cas9 combined with engineered single guide RNA (sgRNA) scaffolds that bind to fluorescent proteins. In this applied science technique, it allows simultaneous imaging of up to seven chromosomal loci in an individual human cell and observes large differences in the chromodynamic properties of different chromosomal loci at a time. This system is a valuable tool for studying the transfiguration of the genome in real time basis, and it is used to label the DNA and then track the movement of DNA in live cells (Ma et al., 2016).

### Summary on Gene Editing Methods

In summary, the CRISPR system allows for site-specific genomic targeting in virtually any organism (Guilinger et al., 2014). It also offers platform that is an efficient way of making precise genetic changes to the human genome. This can be employed for disruption, addition, and correction of genes. Thus, the remaining parts of this review will focus on the utilization of CRISPR tools for understanding the functional genomics of various hematological malignancies, its challenges, as well as the clinical implications.

## Application of Various CRISPR System in Studying the Functional Genomics of AML

AML is a multifaceted genetic disease caused by an interwoven process in which numerous collaborating mutations that allow a cell to evade the checkpoints that normally safeguard it against DNA damage and other disruptions of healthy cell growth (Babaei et al., 2013). It is a type of blood cancer which causes excessive proliferation of abnormal immature leukemic cells known as blasts. Uncontrolled growth occurs through mutations in the FLT3, NPM1, CEBPA, RAS, and c-KIT genes among others. A key gene involved in AML pathogenesis is the FMS-like tyrosine kinase 3, a receptor-type tyrosine-protein kinase (FLT3), positioned at chromosome 13q12 encoding a class III receptor tyrosine kinase that regulates hematopoiesis. This receptor is activated by the binding of the related tyrosine kinase three ligand (FL) to its extracellular domain which induces homodimer formation in the plasma membrane and auto-phosphorylation (Rivera-Torres et al., 2020). The activated receptor kinase phosphorylates multiple cytoplasmic effector molecules in cellular pathways involved in apoptosis, proliferation, and differentiation of hematopoietic cells. Protein kinase activation can be induced by somatic mutations, a common mechanism of tumorigenesis led by the constitutive activation of the receptor resulting in acute myeloid leukemia and acute lymphoblastic leukemia. Advancement in the treatment of leukemia over the last 40 years has impacted and transformed a uniformly fatal disease into one that is somewhat manageable. There are, however, several subtypes of pediatric and adult leukemia that evade treatment and continue poor prognosis; many of these involve FLT3 mutations. For example, the FLT3 ITD associated with a single point mutation in the tyrosine kinase domain is known to induce resistance to tyrosine kinase inhibitors (TKI) treatment (Rivera-Torres et al., 2020).

Many common variants have been associated with hematological traits, but identification of causal genes and pathways have proven to be very challenging. Since acute myeloid leukemia (AML) is a malignant, cancerous development in hematopoietic stem cell lines attributed by several gene mutations, CRISPR serves as an excellent tool to study the underlying molecular and genetic reasons for AML progression. Researchers such as (Rivera-Torres et al., 2020) utilized CRISPR-based site-directed mutation termed as CRISPR-directed mutagenesis (CDM) in deriving plasmid vectors capable of expressing and recapitulating FLT3 sequences mutations as seen in AML patients. The system enabled the *in vitro* study of both the phenotypical cellular changes induced by such FLT3 mutations and the different levels of response to anti-AML drugs, including the potential for discovering new therapeutic targets for AML treatments. A number of researchers have utilized CRISPR systems to investigate the genetic targets behind differentiation blockages commonly seen in causing the development of AML. For instance, Wang *et al.*, identified RNA-binding protein ZFP36L2 as a crucial mechanistic regulator of cell differentiation in AML using cell surface antigen–guided-CRISPR systems (Li et al., 2019; Wang et al., 2021a). Genetic suppression of ZFP36L2 enables myeloid leukemic cells to transit out from an undifferentiated state and resume cellular differentiations. Brabetz *et al.*, gained insights into AML progressions by using CRISPR to functionally study and “edit” the IDH2 genes to both create and correctively repair the mutation, IDH2 R140Q, frequently spotted in AML and are known to impede cellular differentiation and self-repair mechanisms (Brabetz et al., 2017).

Authors such as Ho *et al.*, applied the gene-editing process of CRISPR/Cas systems in knocking out interleukin-1 receptor accessory protein (IL1RAP) genes in human leukemia stem cells in an attempt to abolish relapses of leukemia in AML patients by designing a bone marrow scaffold–mediated delivery of the therapeutic CRISPR system (Ho et al., 2021). Certain studies have utilized CRISPR-mediated gene knockouts in identifying the significant role of KAT7 genes for the survival of AML cells (Au et al., 2021). Tiansu and coworkers attempted to study the development of AML by carrying out a sequential CRISPR/Cas9 gene editing approach involving step-wise introductions of different mutations in induced pluripotent stem cells (iPSC). The technique offered better comprehension of the evolution of AML conditions, highlighted the corresponding changes at transcriptome and chromosome levels between the different stages of diseases progression as well as illuminated potential early disease markers to improve AML prognosis (Wang et al., 2021b).

Combining large-scale or genome-wide genetic analysis with CRISPR and gene targeting yields screening results several folds more accurate with greater gene knockout efficiency and minimal off-target sequence hybridizations. Authors such as Tzelepis *et al.*, incorporated genome-wide CRISPR screening in distinguishing more than 100 essential AML-gene triggers, among which KAT2A was chosen as a promising therapeutic target against AML (Tzelepis et al., 2016). The results revealed that inhibition of KAT2A genes consequently leads to myeloid differentiation and apoptosis, demonstrating its potent anti-AML properties. Wallace *et al.*, identified essential microRNAs which regulate the growth and onset of AML such as miR-155 and miR-150 using large-scale gene knockouts *via* CRISPR/Cas gene editing (Wallace et al., 2016a). Likewise, Lin *et al.*, also employed CRISPR-mediated wide-scale screenings to analyze the genetic dependencies of AML in orthotopic xenograft models, in which several vital AML-linked genetic vulnerabilities were identified such as the SLC5A3 and MARCH5 genes (Lin et al., 2021). *In vitro* CRISPR screens were also utilized to identify genes which sensitizes AML cells to double-negative T cells (DNTs) therapies for effective patient treatments (Soares et al., 2021). Moreover, researchers have also studied the genetic mechanisms that undermine the effectiveness of BCL2 inhibitors in AML treatments using CRISPR-based genome-wide screening (Romine et al., 2021). CRISPR gene knockout screens were also performed to recognize mitochondrial genes necessary for the growth of AML cells. Mitochondrial carrier homolog 2 (MTCH2) was identified as a potent regulator for AML in histone acetylation and subsequent cell differentiation (Khan et al., 2020).

An attempt to alter the abnormal gene function in AML-related disease led researchers to explore therapies that possibly ablate leukemia stem cells (LSCs), thus increasing the chances of eliminating this cancer in patients. A latest study conducted by Tzu-Chieh Ho et al., showcased scaffold-mediated CRISPR-Cas9 delivery system for targeting the gene interleukin-1 receptor accessory protein (IL1RAP) in human LSCs (Ho et al., 2021). This approach was mediated using a bio-reducible lipidoid-encapsulated Cas9/single guide RNA (sgRNA) ribonucleoprotein-induced efficient gene editing. The CRISPR-Cas9 system was an effective strategy for attenuating LSC growth to improve AML therapy. In another approach, a group of researchers from Icahn School of Medicine, Mount Sinia, US have used CRISPR technology in developing a model to study AML at different stages of metastasis. This has paved a way for the group to identify possible biomarkers for early-stage leukemia detection. Hence, CRISPR has not only open the door toward treatment but has also given us an avenue to have huge amount of information pertaining to a disease, in this case it is AML. The information also unraveled other details pertaining to blood cancer myelodysplastic syndrome and clonal hematopoiesis which is often a pre-leukemic condition (Rees, 2021). This has given us a chance to study the molecular mechanisms that underlie the progression of the disease by tracking the evolution of human leukemia. In an another study done by a group of researchers, they have used CRISPR to unveil a new gene that is involved in the regulation of molecular mechanism of leukemia stem cells, the cells responsible for propagating the disease and for therapy resistance. Stau2 has been previously studied in the brain and the nervous system but until now was not known to have a role in cancer and other hematological malignancies (Bajaj et al., 2020).

Acute myeloid leukemia (AML) remains a devastating disease in need of new therapies to improve patient survival. Targeted adoptive T-cell therapies have achieved impressive clinical outcomes in some B-cell leukemia and lymphomas but not in AML. Double-negative T cells (DNTs) effectively kill blast cells from the majority of AML patients and are now being tested in clinical trials. However, AML blasts obtained from ∼30% of patients show resistance to DNT-mediated cytotoxicity; the markers or mechanisms underlying this resistance have not been elucidated. Here, a targeted clustered regularly interspaced short palindromic repeats (CRISPR)/CRISPR-associated protein 9 (Cas9) screen was utilized to identify genes that caused susceptibility of AML cells to DNT therapy. Inactivation of the Spt-Ada-Gcn5-acetyltransferase (SAGA) deubiquitinating complex components sensitized AML cells to DNT-mediated cytotoxicity. In contrast, CD64 inactivation resulted in resistance to DNT-mediated cytotoxicity. Importantly, the level of CD64 expression correlated strongly with the sensitivity of AML cells to DNT treatment. Furthermore, the ectopic expression of CD64 overcame AML resistance to DNTs *in vitro* and *in vivo*. Altogether, the data demonstrated the utility of CRISPR/Cas9 screens to uncover mechanisms underlying the sensitivity to DNT therapy and suggests CD64 as a predictive marker for response in AML patients (Hsu et al., 2013).

## Application of Various CRISPR Systems in Studying the Functional Genomics of ALL

ALL can be characterized by looking at the development of precursor lymphoid into B or T cell. This type of leukemia is usually rare but requires immediate treatment as ALL leukemogenesis is rapid and aggressive (Hsu et al., 2013). Accumulation of lymphoid blast could be observed in patient bearing this disease which is usually translated by enlarged lymph nodes (Kebriaei et al., 2002; Inaba et al., 2013; Litzow and Ferrando, 2015).

The CRISPR/Cas9 knockout system was utilized in the manipulation of ALL’s genomics and in studying the differentiation of ALL into erythrocytes. A study conducted by Xie and group reported that GATA-1 motif flanking 5 kb upstream ARHGEF12 was impaired due to the CC to TT SNPs in hematopoietic cells of ALL patients suggesting that GATA-1 transcriptional activation of ARHGEF12 is crucial in driving erythrogenesis in which ALL patients lacked. The luciferase reporter assay confirmed GATA-1 interaction onto the ARHGEF12 flanking region when compared to the impaired motif brought about by the CC to TT SNPs. To further ascertain that this switch was responsible in driving erythropoiesis, the group employed CRISPR/Cas9 knockout where guide RNA was designed to be specifically targeting the ARHGEF12 gene directly. Result showed a stunted production of red blood cells and immature erythrocytes accumulation (Terwilliger and Abdul-Hay, 2017).

Furthermore, blockade of transcriptional machinery *via* the use of CRISPR system was also demonstrated when the MYB-binding site located proximally upstream of LMO2 was targeted using Cas9. In this study, a second binding motif of MYB was found to occur on LMO proximal site, this occurs due to the translocation event of the LMO2 gene which shift the MYB-binding site to a second MYB-binding site thus duplicating the MYB motif resulting in an increase in LMO2 activation by MYB. Interesting enough, Cas9 cleavage of the duplicated MYB motif resulted in a significant reduction in LMO2 expression (Xie et al., 2020).

ETV6/RUNX1 fusion oncogene commonly found in ALL was also successfully targeted using CRISPR/Cas9. In this case, guide RNA was designed to target the specific ETV6 and RUNX1 fusion region. Subsequent qPCR reported a reduction in the mRNA of ETV6/RUNX1 fusion construct following CRISPR/Cas9–mediated knockout of the fusion region. Furthermore, functional studies of the ETV6/RUNX1 abrogated REH cells, and ALL cell line showed a reduction in proliferative potential which also translates to a reduction in chemoresistance where REH cells were shown to be more apoptotic following chemodrug treatment (Rahman et al., 2017). Moreover, it was also reported that the BCR-ABL fusion gene was successfully targeted in Philadelphia positive ALL cells. Small guide RNA was designed to target a specific region of the fusion protein; this group used the nickase system of CRISPR/Cas9 where cleavage occur between the exon–exon junction, and this resulted in total abolishment of the fusion protein trailed by rapid apoptosis of ALL cells (Montaño et al., 2020).

Derivative of the Cas9 knockout system, the dCas9 repressor, was also used in studying the leukemogenesis of ALL. A study sought to look into the function of ARID5B in ALL resistance toward treatment. The dCas9-KRAB repressor system was used to repress ARID5B, and the knockdown showed a significant increase in IC50 following MTX (chemodrug) treatment, suggesting that ARID5B could probably be downregulated in ALL for chemoresistance. This was interesting as patients with relapse showed significant downregulation of ARID5B (Tan et al., 2020). This study reflects the usage of the CRISPR system in studying the functional genomics of disease progression.

Additionally, ALL metabolism dysregulation was also studied in further understanding the mechanism of ALL leukemogenesis, and it was reported that the PAX5 transcription activator was deactivated in which this trailed increase in glucose uptake of ALL thus supporting ALL development. Using the CRISPR activation system (VP64), the same group induced PAX5 expression which in turn would activate PAX5 repressed gene; this attenuated glucose uptake thus restoring the gate keeping function of PAX5 as a metabolic switch (Xu et al., 2020).

## Application of Various CRISPR Systems in Functional Genomics of CML

This type of leukemia can be characterized by looking at accumulation of granulocyte within the bone marrow. Trailing myeloid precursor aberration neutrophilic and basophilic cell accumulation could also be observed within the peripheral blood. Synonymous to CML is the BCR-ABL fusion oncoprotein resulting from the Philadelphia chromosome, which is a direct result of translocation between chromosome 9 and 22 (Trela et al., 2014; Chan et al., 2017; Chopade and Akard, 2018; Hsieh et al., 2021).

The use of CRISPR in CML is mainly on aberating the BCR-ABL fusion tyrosine kinase. BCR-ABL fusion resulted in a surface protein with constitutive tyrosine kinase activity which is one of the main factors in CML leukemogenesis (Russo et al., 2020; O'Dwyer et al., 2002). Although imatinib mesylate (IM), a first of its kind tyrosine kinase inhibitor, proved to be effective in abrogating CML, patients still suffer relapse prompting scientist to directly target the fusion construct in treatment of CML (Guilhot, 2004; Barnes et al., 2005; Chan et al., 2017).

An interesting study sought to abolish the region of fusion by specifically targeting the ABL exon 6. In this case, the CRISPR knockout system was used where sgRNA were designed to nick a region of about 100 bp on ABL locus within the genome. The CRISPR nickase system was proven to be effective as expression of the BCR-ABL mRNA reduced to only 2.6%, with a total abolishment of protein translation. This also leads to an increase in apoptosis when compared to wild type CML cells. Furthermore, the group also targeted leukemic stem cells (LSCs) using the same system and found out that mice with LSCs-CRISPR–containing engraftment showed a reduction in CML oncogenic properties and restoration of multipotency of LSC with unbiased development toward only myeloid lineage (Sharma et al., 2010).

Recently, the CRISPR/Cas9 system was fused with an *E. coli* exonuclease 3; this resulted in an efficient cleavage of large genomic locus which is more efficient than CRISPR nickase. In CML, this technology was used to target the BCR-ABL gene where an efficient deletion of the target site was observed, and this was also followed by an increase in apoptosis in CRISPR-EXO3–targeted CML cell line (Vuelta et al., 2020). The same group also used the technology to target BCR-ABL *in vivo* where animal models with CRSPR-EXO electroporation showed an increase in survivability and tumor size reduction (Vuelta et al., 2020).

Another example on the use of the CRISPR system in CML can be seen in a study by Cheng and colleagues where Cas9 was designed to target specifically MBD2. MBD2 protein associates with a highly methylated region resulting in gene suppression of crucial factors in proper cellular development. This protein was found to be upregulated in the CML crisis phase. Targeting the MBD2 gene *via* Cas9 resulted in a significant ablation in proliferative potential of CML cell lines (Cheng et al., 2018). Furthermore, *in vivo* mice model also showed reduction in tumor volume; thus, this study proved the applicability of Cas9 in targeting methylation–associated factors in leukemic aberration (Lainšček et al., 2020).

Recently, the CRISPR/Cas9 system has been used to correct the ASXL1 homozygous nonsense mutations that are present in the CML cell line KBM5, which lacks ASXL1 protein expression. This resulted in protein re-expression with normal functionality restored including downregulation of Polycomb repressive complex two target genes (Kim et al., 2014).

The CRISPR/Cas9 system was also used to directly target miRNA locus in CML. Recent research by Arya et al. showed that CRISPR knockout of miR182-5p sensitized CML cells toward TKI treatment. miR182-5p was found to be highly expressed in resistant CML and that it was also associated with improper blast differentiation. Cas9-mediated knockout of miR182-5p relieved leukemic properties allowing for CML to be effectively treated (Arya et al., 2018).

## Application of Various CRISPR Systems in Functional Genomics of CLL

Chronic lymphocytic leukemia is a type of malignancy involving the accumulation of CD5^+^ B cell within the brain, marrow, and lymphoid tissue. This type of leukemia was found to be one of the most occurring type of leukemia, and depending on the degree of mutation-driven proliferation, treatment of this disease varies in which in some patients treatment required is minimal, while in others immediate treatment is required, which reflect the heterogeneity of this disease (Julio Delgado et al., 2020; Milne et al., 2020). Mutation that are usually looked into to determine the degree of the disease is the IGHV mutation, which could form fusion with the BCL family due to translocation, and the use of the B-cell receptor as a target, therefore, favor CLL management (Kipps et al., 2017).

The use of CRISPR in CLL recently focused on driver mutation contributing to CLL leukemogenesis. One of the gene of interest looked upon is the *NOTCH1* gene as it was found to affect CLL homing into the spleen and the brain, which is a major contributor to bad prognosis when the gene is mutated. Using CRISPR/Cas9 targeting, the NOTCH1-PEST protein domain in Mec-1 a CLL cell line showed low levels of DUSP22 expression which significantly correlates with the spleen and the brain homing suggesting that NOTCH1 mutation occurs specifically on the PEST domain which could act as a major driver in CLL leukemogenesis with bad prognosis (Arruga et al., 2017).

In a more recent study, NOTCH1 dysregulation accompanying FBXW7 mutation in CLL was found to be more prominent in patients with worse outcome. FBXW7 is a negative switch of NOTCH1 where physical interaction between these two would deactivate NOTCH1. Furthermore, the group also found that mutation in the WD40 domain of the FBXW7 is common in CLL patients. The use of Cas9 to abrogate the WD40 domain further support that this mutation is a contributing driver in CLL progression into worse prognosis as an increase in NOTCH1 activation was observed despite physical interaction (Close et al., 2019).

In order to further understand CLL progression, a group chose to look into specific CLL mutation, namely, CLL with del (11q) with either TP53 or ATM mutation. Using CRISPR/Cas9, the group induced deletion of the 11q arm and designed small guide RNA against TP53 and ATM. Results showed that del (11q) with TP53 mutation confer leukemic survivability suggesting that these two mutational events co-occurrence potentiate CLL progression. However biallelic mutation of ATM and TP53 result in reduction in CLL engraftment *in vivo* suggesting that mutual targeting of both ATM and TP53 is favorable in aberrating CLL. Further study also confirms the reliability of this method as TP53 and ATM mutation never co-occurred in patient with CLL (Quijada-Álamo et al., 2021a). Targeting of CLL with del (11q) and ATM mutation was also proven to be more effective with BCR or PARP inhibitors, thus further supporting that direct targeting of both TP53 and ATM is favorable in CLL aberration (Quijada-Álamo et al., 2020).

## Application of Various CRISPR Systems in Functional Genomics of Other Hematological Malignancies

CRISPR has also been utilized to study the functional genomics of other types of hematological malignancies other than AML, ALL, CML, and CLL. Other common hematological malignancies are multiple myeloma, Hodgkin, and non-Hodgkin lymphomas. Researchers usually conduct whole genome sequencing (WGS) to identify crucial genes. Next, they utilized CRISPR to screen the genes identified with WGS to uncover the function of the genes and how it contributes to the malignancies. CRISPR is also used to uncover how the malignancies adapt and develop resistance to cancer treatments. It is hoped that by studying the functional genomics, new treatment targets against these malignancies can be identified and new treatment options can be developed, tabulated in Table.1.

**TABLE 1:** Recent findings on CRISPR usage in targeting leukemia (Ergo 2015–2021).

Type of cas/Tools	Gene target	Leukemic type	Target method	Outcome	References
Cas9/Genomic cleavage	USO1	B-ALL with MLL translocation	Knockout of gene	Deregulated mTOR signaling and reduced colony-forming potential	Jaiswal et al. (2021)
Cas9/Genomic cleavage	LMO2 promoter	T-ALL	Knockout of transcription factor binding site	Blocks binding of crucial TFs reducing LMO2 oncogene expression	Xie et al. (2020)
Cas9/Genomic cleavage	BCR-ABL	BCR-ABL positive ALL	Ablation of the BCR-ABL breakpoint and SH2 kinase domain	Delayed leukemic onset post transplantation in mice models	Montaño et al. (2020)
Cas9/Genomic site nicking	EZH2	T-ALL	Knockout of gene at exon 2	Sensitizes ALL cells toward chemodrug treatment in the primary model	León et al. (2020)
Cas9/Genomic cleavage	LDHA	T-ALL	Knockout of gene	Arrested cell growth and suppression of the oncogene C-MYC in the primary model	Yu et al. (2020)
Cas9/Genomic cleavage	microRNA-21	CML	Knockout of miRNA locus	Inhibition of cellular proliferative potential and increased CML sensitivity toward Imatinib treatment	Zhang et al. (2021)
Cas9/Genomic cleavage	BCR-ABL	CML	Knockout of BCR-ABL junction	Inhibition of proliferation *in vitro* and *in vivo* xenografted transplant models	Chen et al. (2020)
Cas9/Genomic cleavage	RUNX1	CML	RUNX1 gene knockout	Sensitizes CML toward glucocorticoids and mTOR, BCL and VEGFR inhibitors, and increased *ex vivo* CAR-T cells targeting sensitivity	Adnan Awad et al. (2021)
Cas9/Genomic cleavage	BCR-ABL	CML	BCR/ABL gene knockout	Improved delivery and targeting of CRISPR system *via* delivery by PEG nanoparticle, improved survival of mouse models	Liu et al. (2018)
Cas9/Genomic cleavage	HDAC1/2	CML	HDAC1/2 gene knockout	Induced cellular apoptosis post knockout of imatinib-resistant patient-derived CML	Chen et al. (2019)
Cas9/Genomic Knock in	ASLX1	CML	Wild-type ASLX1 was used at template for HDRF-mediated insertion	Reduced CML model cell growth and induce differentiation increased overall survival of mice xenotransplanted with knocked in CML model lines	Valletta et al. (2015)
Cas9/Genomic Knock in	TOP2α	CML	Insertion of GAG//GTAA **AC** →GAG//GTAA **GT** to exon 19/intron 19.5 splice site of TOP2α	Increased etoposide induced DNA damage otherwise desensitized in CML with suboptimal TOP2α	Hernandez and Carvajal-Moreno, (2021)
Cas9/Genomic cleavage	APOBEC3	CLL	Direct gene knockout and cis regulatory element deletion	APOBEC3 expression occurs in synergy with NFATc1 enhancer binding and BCR pathway post BTKi treatment leading to genomic instability	Wang et al. (2021c)
Cas9/Genomic cleavage	BIRC/del (11q)	CLL	Direct gene knockout	Uncovered mechanism on BIRC/del (11q) on CLL leukemogenesis where primary model with both BIRC1 mutation and 11q deletion was sensitized to venetoclax	Quijada-Álamo et al. (2021b)
Cas9/Genomic cleavage	TP53	CLL	Direct p53 gene knockout	P53 knockout reveals multiple p53 dependent miRNA crucial for CLL development	Blume, (2015)
Cas9/gRNA transcription factor pool cleavage	147 CLL Transcription factor library and PAX5	CLL	Transcription factor gene knockout	PAX5 was found to be predominantly contributing to CLL progression in primary samples where reduced CLL cell growth was observed	Ott et al. (2018)
Cas9/Genomic cleavage	Notch2	CLL	Direct gene knockout	Notch2-deleted cells deactivated Wnt signaling leading to the impairment in tumor survival	Mangolini et al. (2018)
Cas9/Genomic cleavage	Genome wide	ABC DLBCL	Whole genome knockout	In ABC DLBC, EBF1, IRF4, CARD11, MYD88, and IKBKB knockout reduced viability	Reddy et al. (2017)
		GCB DLBCL		In GCB DLBCL, the knockout of ZBTB7A, XPO1, TGFBR2, and PTPN6 reduced viability	
Cas9/Genomic cleavage	Genome wide	GCB DLBCL	Whole genome knockout	Discovered the synthetic lethal interaction between CREBBP and EP300 genes in DLBCL.	Nie et al. (2021)
Cas9/Genomic cleavage	Genome wide	ABC DLBCL	Whole genome knockout	Discovered a new form of BCR signaling coordinated by multiprotein supercomplex formed by MYD88, TLR9, and BCR (My-T-BCR) in ibrutinib-responsive DLBCL.	Phelan et al. (2018)
		GCB DLBCL			
Cas9/Genomic cleavage	Foxp1	Mice model of B-cell lymphoma	Knockout of gene	Knockdown of Foxp1 causes upregulated cell surface I-Ab (MHC-II) expression and restores immune surveillance	Felce et al. (2020)
Cas9/Genomic cleavage	Genome wide	BL	Whole genome knockout	The genes IGLL5, BACH2, SIN3A, and DNMT1 were determined to be involved in tumorigenesis of BL. ID3 is the most frequently silenced gene in all subtypes of BL.	Panea et al. (2019)
Cas9/Genomic cleavage	Sp3 and Phip	Eμ-Myc genetically engineered mouse model of BL	Knockout of genes	Both Sp3 and Phip act as tumor suppressors in Eμ-Myc driven lymphomas	Katigbak et al. (2016)
Cas9/Genomic cleavage	VPREB1	MM	Knockout of gene	Knockout of VPREB1 on primary MM cells resulted in reduction of myeloma cell proliferation	Khaled et al. (2021)
Cas9/Genomic cleavage	MUC1-C	MM	Knockout of gene	It was discovered that MUC1-C activates MYC gene through a TCF4-mediated mechanism	Tagde et al. (2016)
Cas9/Genomic cleavage	Genome wide	MM	Whole genome knockout	Proteasome regulatory subunit PSMC6 confers bortezomib resistance	Shi et al. (2017)
Cas9/Genomic cleavage	Genome wide	MM	Whole genome knockout	Drug resistance in MM was associated with E3 ligase complex genes, PCDHA5, ANKMY2, RB1, CDK2NC, and TP53	Bohl et al. (2021)
				Inactivation of ATM, FANCA BRCC3, and RAD54B made MM cells more sensitive to chemotherapy	
dCas9/Genomic interference and activation	Genome wide	MM	Whole genome	Knockdown of HDAC7 and SEC61A increased the levels of BCMA	Ramkumar et al. (2020)
Cas9/Genomic cleavage	Genome wide	TCL	Whole genome knockout	Discovered JAK2 and IKZF1as potential treatment targets. In TP53-wild-type TCLs, MDM2, and MDMX can be blocked with ALRN-6924	Ng et al. (2018)
Cas9/Genomic cleavage	600 genes associated with PD-L1	ALCL	Knockout of gene	Discovered that PD-L1 is induced by STAT3 and GRB2/SOS1	Zhang et al. (2019)
Cas9/Genomic cleavage	Genome wide	PEL	Whole genome knockout	Inactivation of UBE2G1 conferred resistance against LEN and POM, while inactivation of CRBN provided resistance to CC-122	Patil et al. (2019)
Cas9/Genomic cleavage	Genome wide	AML	Whole genome screening	492 AML-specific genes, including DOT1L, BCL2, and MEN1. KAT2A inhibition demonstrated anti-AML activity by inducing myeloid differentiation and apoptosis, suppressed the growth of AMLs	Tzelepis et al. (2016)
Cas9/Genomic cleavage	IL1RAP (interleukin-1 receptor accessory protein)	AML	Knockout of gene	IL1RAP knockout reduced LSC colony-forming capacity and leukemic burden	Ho et al. (2021)
Cas9/Genomic cleavage	RBM39 (RNA-Binding Motif Protein 39)	AML	Knockout of gene	Effects of RBM39 loss resulted in lethality of spliceosomal mutant AML, providing a strategy for treatment of AML.	Wang et al. (2019)
Cas9/Genomic cleavage	miR-155	AML	Whole genome screen	CRISPR-Cas9 global loss-of-function screen to simultaneously test the functions of individual miRNAs and protein-coding genes. miR-155 was promoting cellular fitness, which was confirmed with 2 distinct miR-155 targeting by CRISPR-Cas9 lentiviral constructs	Wallace et al. (2016b)

DLBCL is the most common non-Hodgkin lymphoma worldwide and is a fast growing cancer which affects the B lymphocytes (Diffuse Large B-Cell Lymphoma, 2021). DLBCL have been categorized into three subgroups: activated B cell–like [ABC], germinal-center B cell–like [GCB], and unclassified based on their gene expression profile (Chapuy et al., 2018). Large scale genetic profiling studies have also shown that there are many recurrent altered genes in DLBCL (Reddy et al., 2017; Schmitz et al., 2018); however, the function of the genes and its contribution to lymphomagenesis is not well understood. Therefore, CRISPR has been utilized in multiple studies to better understand how the genetic alterations interact and leads to tumorigenesis. In a study by Reddy et al. the researchers utilized CRISPR to identify crucial oncogenes in DLBCL and then compared it to the expression profile of 1001 DLBCL patients. Based on the analysis, they determined a list of crucial oncogenes that act as genetic drivers for DLBCL (Reddy et al., 2017). In the study, 35 driver genes were identified as functional oncogenes as their knockout with CRISPR resulted in reduced viability of DLBCL cells. Nine out of the genes were then found to be important in specific subtypes. In the ABC DLBCL subtype, EBF1, IRF4, CARD11, MYD88, and IKBKB knockout produced significant reduction in cell viability, whereas in GCB DLBCL, the knockout of ZBTB7A, XPO1, TGFBR2, and PTPN6 was more selectively lethal to the subtype. Based on the results obtained, the researchers postulate that these genes can be utilized to classify the different subtypes of DLBCL and also be specific treatment targets for future treatment of different subtypes of DLBCL. In another recent study by Nie et al. the researchers conducted genome-wide CRISPR screens on the DLBCL cell line: RC-K8 and discovered the synthetic lethal interaction between CREBBP and EP300 genes (Nie et al., 2021). In this study, a genome-wide CRISPR-Cas9 loss-of-function screening determined CREBBP and EP300 as crucial genes. It was determined that DLBCL cells that have deficiency in both CREBBP and EP300 are sensitive to histone acetyltransferases inhibition, and this can be utilized as a treatment target in the future.

Phelan et al. tried to determine the cause of ibrutinib sensitivity in certain DLBCL cell lines. Based on their genome-wide loss-of-function CRISPR-Cas9 screens on three ibrutinib-sensitive ABC cell lines, one ibrutinib-insensitive ABC cell line, and four ibrutinib-insensitive GCB cell lines, they uncovered a new B-cell receptor signaling in ibrutinib-responsive cell lines which is coordinated by a multiprotein supercomplex formed by MYD88, TLR9, and the BCR (My-T-BCR supercomplex) (Phelan et al., 2018). There are also *in vivo* researches utilizing CRISPR to elucidate the function of genes in B-cell lymphomas. Felce et al. conducted research on murine A20 lymphoma cell line which is B-cell lymphoma derived from mice as a model for B-cell lymphoma. The researchers utilized CRISPR to knockdown the Foxp1 expression in the cells which upregulated cell surface I-Ab (MHC-II) expression without impairing cell viability *in vitro*. The reduction of Foxp1 restores immune surveillance. This shows that Foxp1 helps the lymphoma cells evade the immune system. Based on the results, it is hoped that targeting Foxp1 in B-cell lymphomas will enhance the effects of other immunotherapies. By utilizing CRISPR in tandem with other molecular techniques, researchers were able to detect and determine the function of genes crucial in DLBCL (Felce et al., 2020).

Burkitt’s lymphoma (BL) is another type of non-Hodgkin lymphoma. It is divided into three subtypes which are sporadic, endemic, and immunodeficiency associated (Lenze et al., 2011). Panea et al. conducted whole genome sequencing (WGS) and transcriptome sequencing on 101 patient samples to identify the genomic basis for Burkitt’s lymphoma. CRISPR was done to functionally annotate the role of oncogenic drivers that were identified from WGS. The genes IGLL5, BACH2, SIN3A, and DNMT1 were determined to be involved in the tumorigenesis of BL. In this study, they also identified ID3 as the most frequently recurrently silenced gene in all subtypes of BL. Further *in vitro* and *in vivo* study uncovered the role of ID3 in deregulation of TCF3 and TCF4 which affects the cell proliferation in BL cells (Panea et al., 2019). Katigbak et al. conducted CRISPR on an *in vivo* model of Burkitt’s lymphoma: Eμ-Myc genetically engineered mouse model (GEMM). The results of this study shows that both Sp3 and Phip behave as tumor suppressors in Eμ-Myc driven lymphomas (Katigbak et al., 2016). Both these studies utilized CRISPR to identified and validate tumor suppressor genes *in vitro* and *in vivo* from BL genome sequencing data.

Multiple myeloma (MM) is the cancer of the plasma cells and is relatively rare cancer that contributes around 1.8% of all diagnosed cancer in United States (Myeloma, 2021). There are limited treatment options for MM, and relapse and developing resistance against treatment drugs is a worrying trend in MM (Anderson et al., 2009). Therefore, CRISPR has been utilized to identify crucial genes that can be targeted as a treatment option and also to determine genes that confer resistance to MM to allow for personalized treatment of MM patients. Khaled et al., 2021 utilized CRISPR to knockout crucial gene in primary MM cell to reduce its viability. After conducting a bioinformatic analysis on the gene expression data of MM cells, they identified V-set pre B-cell surrogate light chain 1 (VPREB1) gene as the knockout target gene. CRISPR-Cas9–mediated knockout of VPREB1 on primary MM cells was done, and it resulted in reduction of myeloma cell proliferation (Khaled et al., 2021). The results show that VPREB1 is a crucial gene that affects proliferation in MM cells and can be a possible target for treatment of MM. In another study, Tagde et al. tried to uncover the mechanism by which MYC oncoprotein is upregulated in multiple myeloma. MYC is a crucial oncoprotein for MM cells and plays an important role on the progression and survival of MM. However, there is no comprehensive research that shows how MYC is upregulated in MM cells. CRISPR was utilized to silence mucin 1 C-terminal subunit (MUC1-C) which led to reduction in MYC expression levels. The result was further validated by looking at the expression levels in primary cells from MM patients. It was discovered that MUC1-C activates MYC gene through a TCF4-mediated mechanism (Tagde et al., 2016). By utilizing CRISPR to silence genes, the pathway on how MYC expression is upregulated in MM cells was discovered.

In another study by Shi et al., the researchers tried to uncover the pathway of bortezomib resistance in MM cells. They started by doing CRISPR targeting the 19,052 human genes in the MM cell line: RPMI8226 and selecting the cells in lethal doses of bortezomib. The surviving cells were propagated, and the genome was sequenced to identify inactivated genes that led to resistance. Furthermore, CRISPR was done to validate the function of the genes, whereby proteasome regulatory subunit PSMC6 was the only gene validated to reproducibly confer bortezomib resistance (Shi et al., 2017). The researchers therefore determined that PSMC6 is crucial in bortezomib resistance in MM cells. Further research showed that the ability of bortezomib to inhibit chymotrypsin-like proteasome activity was severely affected in cells deficient in PSM6. Bohl et al. performed combined whole-exome sequencing (WES) on patient samples and relapsed patient samples. Based on the comparison between the samples, 170 relapse-specific mutations were identified and CRISPR was performed against them to determine their function. Based on the CRISPR results, 15 of them are functionally linked to drug resistance. In this study, it was discovered that there were specific genes associated with resistance against each type of drug. Resistance against lenalidomide was associated with E3 ligase complex genes, dexamethasone was associated with PCDHA5 and ANKMY2, bortezomib was linked with RB1, and CDK2NC and melphalan were linked with TP53 (Bohl et al., 2021). It was also discovered that inactivation of genes such as ATM, FANCA, BRCC3, and RAD54B, which are involved in DNA damage repair, made MM cells more sensitive to chemotherapy. The findings from this study suggest that gene alteration is linked to sensitivity or resistance to chemo drugs which can be utilized in future to advice treatment options for MM patients. Ramkumar et al. wanted to identify pathways that control the expression of B-cell maturation antigen (BCMA) since in one of the ways MM develops resistance against immunotherapy is *via* loss of surface antigen expression. In the study, CRISPR interference and CRISPR activation were used to identify genes that led to increase in BCMA on the MM cell surface. They discovered that the knockdown of HDAC7 and SEC61A (part of SEC61 complex) increased the levels of BCMA (Ramkumar et al., 2020). They then verified their results by using drugs to inhibit HDACs protein and the Sec61 complex, whereby an increase in BCMA levels was observed. The cells with elevated BCMA were also more susceptible to the immunotherapy drug: BCMA-targeted antibody–drug conjugate (ADC), HDP-101. These studies show how CRISPR has been utilized to uncover underlying genomic changes in MM cells that confers them resistance against treatment. The same information can then be used to develop new treatment options for patients.

CRISPR has also been used to uncover the functional genomics in rare hematological malignancies. Ng et al. conducted a study on T- and NK-cell lymphomas (TCL) which have poor clinical outcomes. The goal of the study was to identify targetable vulnerabilities in TCL using genome-wide CRISPR screening. They discovered that potential treatment targets JAK2 or IKZF1 which can be targeted with available inhibitors (Ng et al., 2018). In TP53-wild-type TCLs, they discovered MDM2 and MDMX as vulnerable targets, whereby their interaction with p53 can be blocked with ALRN-6924. This study identified treatment targets in TCL where there are available drugs. In another study, Zhang et al. conducted CRISPR on anaplastic lymphoma kinase (ALK)–positive anaplastic large-cell lymphoma (ALK + ALCL) to uncover programmed cell death protein 1 (PD-L1) regulation (Zhang et al., 2019). They discovered that PD-L1 is induced by STAT3 and GRB2/SOS1. STAT3 and GRB2/SOS1 through action of IRF4 and BATF3 transcription factors act on enhancer region of PD-L1 gene to induce its expression. By uncovering the pathway of PD-L1 induction, new treatment targets can be identified to allow for improved immunotherapy strategies. Patil et al. conducted a study to uncover the toxicity of cereblon-modulating agents (CMs) on primary effusion lymphoma (PEL). CMs are drugs used to treat PEL, and the downstream mechanisms of toxicity are not well studied. The researchers conducted genome-wide CRISPR selection screening against CMs with increasing toxicity in PEL: lenalidomide (LEN), pomalidomide (POM), and CC-122 (Patil et al., 2019). Based on the results they determined that inactivation of the E2 ubiquitin–conjugating enzyme UBE2G1 conferred resistance against LEN and POM, while inactivation of CRBN provided resistance to CC-122. The genes can be utilized in future as biomarkers to determine treatment options for patients.

Despite improved responses with frontline therapies in lymphoma relapsed and refractory cases remain a significant challenge. Genome-editing technology has become a potential therapeutic option in lymphoid malignancies although most applications of CRISPR-Cas9 to date have been limited to bench studies. Recently, an exciting study unveiled the target gene for the therapeutic activity of NUTLIN3A, a novel small-molecule antagonist of MDM2 that promotes TP53 activation. Using mouse models lacking TP53 target genes, the authors demonstrated that *BBC3* (*PUMA*) is responsible for the resistance of NUTLIN3A in lymphomas. Furthermore, CRISPR-Cas9–mediated silencing of the *BBC3* gene confirmed that BBC3 expression might predict NUTLIN3A treatment outcomes in patients. More recently, a study reported the application of a CRISPR-Cas9 system to disrupt *CXCR4* expression in mantle cell lymphomas (MCL), a highly aggressive subset of B-cell non-Hodgkin lymphomas (NHLs). Performing lentiviral-based CRISPR-Cas9–mediated silencing of *CXCR4*, the researchers found that reactive oxygen–mediated CXCR4 expression is a key signal inducing autophagy, which contributes to the survival of bortezomib-resistant MCL cells (Zhang et al., 2016).

## Challenges in Using CRISPR Tools in the Study of Functional Genomics

While CRISPR-Cas9 technology has emerged as a promising tool in interrogation of gene functions, it has been recognized that several technical challenges greatly mar the progression of this field. Some of the more perplexing issues have been encountered through the use of CRISPR in pooled screening, occurrence of off-targets, false positive and false negative results, low efficiency of HDR events, use of high passage number cell lines, and limitations to the types of genes that can be studied.

One of the major disadvantages in CRISPR, which has been identified early in the development of this technology, is the potential to generate off-target double-strand cuts. Although the typical sgRNA which consists of 22bp is able to provide high enough diversity for it to remain unique even within the human genome of over three billion bp, many studies have demonstrated that a degree of base mismatch is well tolerated by the Cas9 system, leading to the cleavage of alternative sites (Hsu et al., 2013; Lin et al., 2014). Furthermore, it has also been shown that if these mismatches are to occur further away from the PAM sequence, the likelihood of binding and cleavage would be higher (Hsu et al., 2014). Moreover, the presence of minor DNA or RNA bulge resulting from insertions or deletions in the genome are also tolerated (Lin et al., 2014). Some solutions to this problem has been devised including development of predictive scores in guide RNA design software (Tycko et al., 2016) and also incorporating the use of enhanced specific Cas9 (eSpCas9) and high fidelity Cas9 (SpCas9-HF1) (Kleinstiver et al., 2016; Slaymaker et al., 2016). Alternatively, Cas9 can also be delivered in a protein form to provide immediate activity and degrade shortly thereafter to prevent binding and cleavage of other sites (Kim et al., 2014). A recent study has further demonstrated that the precision of CRISPR editing can be significantly enhanced with the incorporation of a hairpin sequence at the 5’ end of the sgRNA (Kocak et al., 2019).

Another major hindrance lie in the shortcomings encountered when pooled library screens are performed, whereby paracrine signaling from wild-type cells may mask the effects of knockout (KO) cells (Ford et al., 2019). This is exemplified in growth factor KO cultures where continued compensatory secretion by adjacent unaffected cells is able to prevent the emergence of the true phenotype. Therefore, pooled library screens of heterogenous cultures may fail to identify the full set of genes responsible for particular phenotypes. Additionally, another disadvantage of pooled screening is that the range of phenotypes that can be read is restricted, typically only to survival and proliferation studies. Some initial efforts made in this area have been able to partially address the abovementioned limitations. FACS, for example, has been shown capable of enriching cultures of positively transformed cells *via* expression of fluorescence proteins and also cell surface markers, which have been demonstrated even in patient-derived xenografts (Hulton et al., 2020). This would allow for more precise investigations of cells by exclusion of crosstalk by neighboring wild-type cells.

Another major concern is that genes which are essential for survival cannot be interrogated *via* complete knockout (Ford et al., 2019). In such cases, it would be suitable to employ CRISPR interference for knockdown studies. On the other hand, it would be appropriate to utilize KO approaches to investigate genes which are capable of maintaining function at low expression levels considering the phenotype could be misinterpreted with knockdown studies. These circumstances would need to be identified prior and addressed with the use of appropriate CRISPR systems to avoid the emergence of perplexing outcomes.

Furthermore, CRISPR systems have been known to generate false positive and false negative results. False positives can potentially arise when genes with high copy number or genes amplified in aneuploid cells are targeted. In this instance, the double-strand breaks that occur at numerous sites cause the cells to undergo apoptosis, which leads to false positive results and hence misidentification of cancer survival genes. This has been shown by several groups which studied sgRNAs on amplified genes in malignant cell lines (Aguirre et al., 2016; Munoz et al., 2016; Sheel and Xue, 2016). A straightforward way of identifying this scenario is when apoptosis of cells occurs independent of gene of interest (GOI) transcriptional arrest. This scenario has been exemplified in knockout studies of the high copy number BCR-ABL fusion gene found in Philadelphia chromosome–related CML (Wang et al., 2015). A possible solution to overcoming this problem would be using CRISPRi to repress transcription of the GOI without introducing double-strand breaks to the genome. In terms of false negatives, these scenarios are most often seen with the use of sgRNAs that exhibit low relative activity. To overcome this predicament, investigators could employ sgRNAs which have been prior validated by other research groups in wet lab experiments. With the advent of machine learning, it is also possible to design sgRNA with higher activity based on published sequences.

In the use of homology-directed repair (HDR) to introduce foreign GOIs into the human genome, low efficiency has been a major obstacle. Knocking-in genes with CRISPR/Cas9 is able to circumvent the detrimental effects on cellular phenotype caused by random integration with lentiviral systems. This approach has many potential uses including the possibility of creating disease models by incorporating mutant genes and also determining the functions of promoters and repressors in knock-in studies. Studies aimed at increasing HDR events have investigated approaches such as using blocking mutations through the incorporation of silent mutations in the PAM and target gene sequence to prevent re-cutting by the Cas9 nuclease once the donor DNA has been ligated (Paquet et al., 2016). Another strategy is *via* the engineering of a fusion fork head protein homolog1 transcription factor (Fkh1p) and LexA DNA-binding protein to generate LexA-Fkh1p, which is capable of efficiently recruiting exogenous DNA to the cleavage site (Roy et al., 2018). Furthermore, as it has been elucidated that HDR takes place mainly during the S and G2 phase of the cell cycle, a study was conducted to cell-cycle-tailor the expression of Cas9 (Gutschner et al., 2016). This delay in expression until the initiation of the S and G2 phases was achieved *via* fusion of Cas9 to the N-terminal of human geminin and was able to increase HDR events up to 87%.

Currently, researchers are also highly reliant on cell lines to conduct pooled screenings. Doubts have been cast on the ability of cell line studies to correlation with actual human diseases especially involving functional genomics as cultures maintained for prolonged durations accumulate point mutations, acquire epigenetic modifications, and also develop chromosomal aberrations (Rebuzzini et al., 2015). The ideal scenario would be to conduct CRISPR screens on primary cells isolated from human tissues/samples while in low passage culture (Hulton et al., 2020). Optimum growth media should also be selected for maintenance of native cellular physiology as it has been known that cells grown on different media tended to display varying gene expression patterns.

Adequately addressing the abovementioned issues will greatly enhance the utility of CRISPR and broaden applications to other emerging areas. Moreover, confounding results occurring from off-targets, false positives, false negatives, and failure of phenotype development could be further prevented to make CRISPR a more robust system in the interrogation of functional genomics. Additionally, it is to be noted that regardless of the hematological malignancies, the potential applications of the CRISPR on gene expression studies remain the same.

## Clinical Implications of CRISPR Applications in Hematological Malignancies

Advancements in CRISPR/Cas9 technology have greatly impacted basic research in hematological malignancies which have immense potential to be translated into clinical applications in the near future. Among the most promising are in the areas of drug discovery, identification of treatment resistance, disease modeling, and genetic manipulation to improve other cancer therapies such as CAR-T cells and editing HSCs for autologous hematopoietic reconstitution.

CRISPR has been extensively explored for discovery of drug targets as potential treatments through genetic screens on cancer cell lines. This approach, among others, has the capacity of identifying essential genes for survival, thereby establishing actionable targets for therapeutic development. A study conducted on AML cell lines, which employed genome-wide CRISPR screening, has identified that survival of the cancer cells is dependent on the mRNA decapping enzyme scavenger (DCPS) (Yamauchi et al., 2018). Further study on inhibitors of DCPS indicated strong anti-leukemic effects by causing mis-splicing of pre-mRNA. Transcription factors (TF) have also been known to be dysregulated in cancers and can be a main vulnerability in its pathogenesis. *PAX5* and *IKZF1* are among the most commonly altered TFs in B-ALL, affecting approximately 80% of patients (Mullighan et al., 2007). CRISPR screens on these TFs were able to identify downstream effectors including *CB2*, *TXNIP*, and *NR3C1*, which are targetable by inhibitors (Xu et al., 2020). This approach, aside from identification of specific genetic vulnerabilities present in cancer cells, is also applicable for delineating the roles of genomic aberrations in affecting cellular fitness which may further provide clues to underlying causes in drug resistance development and how these polymorphic variants drive the growth of cancer (Shalem et al., 2014; Wang et al., 2014; Hart et al., 2015).

In identification of drug resistance genes, a genome-scale CRISPR KO screen has been explored in BCR-ABL1 CML cell lines (Lewis et al., 2020). This approach was found to identify novel drug resistance mechanisms which are related to MAPK signaling and apoptosis *via* the intrinsic pathway. The information generated from such screens could potentially be used to design combinatorial therapies to simultaneously target the resistance and vulnerable survival genes, thereby reestablishing treatment susceptibility.

The absence of cellular models which closely recapitulates disease state in leukemic patients has been a major hurdle in the drug discovery process. Among the most burgeoning issues are the heterogeneity of mutations and non-uniform leukemic cell distribution that co-exist *in vivo*. In these terms, gene editing with the CRISPR system has presented a simple and versatile solution to leukemic disease modeling with the capability of introducing multiple deletions and insertions to simultaneously manipulate expression of several genes and alleles (Lucas et al., 2017). Furthermore, downstream identification of targets for leukemia treatment could also be streamlined with this strategy. This has been exemplified in the generation of a CLL line with complete biallelic loss of the *ATM* gene function to mimic this adverse prognosis state found in approximately 1/3 of patients (Quijada-Álamo et al., 2020). Furthermore, drug screening revealed a previously unknown susceptibility of del (11q)/*ATM*-mutant to inhibition of PARP and BCR. This discovery has unraveled that this high-risk cytogenetic abnormality is treatable with a combination of olaparib and ibrutinib, which confirms the feasibility in utilizing CRISPR in modeling and therapeutic discovery for hematological malignancies.

CRISPR is also able to improve on other cancer therapies including chimeric antigen receptor (CAR) T cells. At present, CAR-T cells have been investigated to treat ALL, CLL, and non-Hodgkin lymphoma through the ectopic expression of CD22, CD20, CD19, and dual B targeting (Paczesny et al., 2018). However, this treatment option is currently limited by low persistence and function of T cells, cytokine release syndrome, treatment-associated toxicity, and labor/cost-intensive preparation processes. To overcome these issues, CRISPR has been utilized to augment CAR-T cell therapy by knocking out endogenous TCR-β which leads to higher efficiency of anticancer activity (Legut et al., 2018). Additionally, it was also found that CRISPR can be used to disrupt T-cell surface receptor inhibitors including PD1 to block immunosuppressive signaling (Xia et al., 2019). Further studies are also underway to develop CRISPR engineered universal allogeneic T cells by knockout of MHC I and TCR, which may allow for upscale productions to reduce associate costs, duration of manufacturing, and the need for highly skilled technical personnel (Ren et al., 2017; Liu and Zhao, 2018).

The mortality rates in the early nineties were more than 80% but have improved gradually to 59% due to multiple reasons. In recent years, autologous HSC transplantation has gained traction with the advent of gene editing technologies which enables the rectification of disease-causing mutations in the patients’ own cells, thereby circumventing complications of allogenic transplants, which includes the lack of suitable donors and the development of graft-versus-host disease. However, earlier studies with viral vectors which integrate randomly into the host genome have raised concerns over activation of oncogenes, leading to secondary leukemia (Zhang and McCarty, 2017). CRISPR/Cas9 is, therefore, able to offer a solution to this predicament with its capability to deliver non-integrative components for site-specific genome editing of *ex vivo* HSC modifications. Additionally, this system is also able to couple with conventional gene therapy to guide donor DNA to safe harboring sites for HDR gene integration. These possibilities open new avenues for researchers to explore not only just silencing and activation of expression but also gene replacement as potential therapies for leukemia. As applications of the former methods are obvious, the latter approach will be highly valued in replacing essential genes involved in complex translocations which gave rise to the hematological malignancy. Early studies on correcting the ASXL1 mutation in the CML cell line KBM5 has already confirmed the restoration of gene function and prolonger survival of xenograft mouse models (Valletta et al., 2015). Furthermore, a publication by Sánchez-Rivera and Jacks in 2015 reported a CRISPR-Cas9 AML mouse model system which has been developed by the Ebert group. It is a lentivirus-mediated *ex vivo* editing of single or multiple genes in a primary mouse HSPCs. This highlights the potential of the CRISPR system for *ex vivo* somatic genome editing of hematopoietic cells, which can be further exploited for various human hematological malignancies.

The applications of CRISPR in hematological malignancies spans across fundamental research to clinical investigations and have made rapid progress in the revelation of vulnerable targets, improvements on other existing therapies, and cellular modifications. In the near future, it is expected that this technology will serve as a practical solution both individually and in combinational therapies to improve the remission rates of leukemic patients.

## Summary and Future Perspectives

This review has clearly demonstrated that CRISPR has served well in deciphering and understanding the genetic basis of hematological malignancies and has bloomed to become the new gamechanger in the field of genome editing by overcoming all the limitations that were displayed by other editing techniques earlier. In spite of all the merits posed by this so-called prime technique, CRISPR also come with certain limitations like its earlier predecessors.

Currently, CRISPR systems can be delivered in different modes whereby, gene editing can be achieved through transient or stable delivery system. Viral-based transfection of CRISPR is the most efficient method for producing stably-modified cells (Lino et al., 2018). Nevertheless, there has been a recent paradigm shift where non-viral methods are becoming the main homestay given biosafety and ethical issues when utilizing viral vectors. Non-viral vector systems include systems such as lipid nanoparticles, cell-penetrating peptides (CPPs), DNA “nanoclews,” and gold nanoparticles. Having said that, the efficiency of the delivery still remains a challenge in hematological-related malignancies. There has been special technique developed to shuttle the cargo of the CRISPR system into the cell *via* rapid cell mechanical deformation (Peyravian et al., 2021). This has, to a certain extent, alleviated the limitation posed earlier and has allowed high delivery efficiency in hematological malignancies.

The issue of specificity in the CRISPR system is a major concern since Cas9 binds to unintended genomic sites for cleavage, termed as off-target effects. The target efficiency of the CRISPR system is only determined through 20 nucleotide sequences of guide RNA (gRNA) and PAM sites adjacent to target loci. If there are more than three mismatches between target sequences and 20 nucleotides of gRNA, it can result in off-target effects. Researchers have proposed two types of off-target effects, the first types of off-target effects likely to occur due to the sequence homology of the target loci and the next types of off-target sites occur in the genome other than the target site (Naeem et al., 2020). The effect causes multiple cellular issues at the genomic level and in turn leads to deletions, alterations in the respective gene, and sometimes could lead to lethal genetic mutations. There are different methods established to validate the off-target effects and the most unique of them all is the Genome-wide Unbiased Identification of DSBs Enabled by Sequencing (GUIDE-seq). This provides an unbiased and genome-wide method for detecting CRISPR RNA-guided DSBs in cells (Kim et al., 2021). This groundbreaking study has increased the practical viability of the off-target detection. With standardization of all the methods that has been drawn over the years, it makes the gene editing tool known as CRISPR to be a possible therapeutic approach in field of hematological malignancy.

The molecular scissors known as “CRISPR” provides complete silencing, and knockdown of gene can still be a valuable means depending on the type of malignancy. This method of gene silencing usually involves an extended time frame to generate a stable cell line. Taking into consideration on the other gene editing tools available with the likes of ZFN, TALENs, and meganucleases, it can be said that CRISPR is the most efficient, stable, and effective. CRISPR is appropriate for delineating gene functions, and genome-editing technology is the apparent option for creating a true genetic null allele, introducing a point mutation, and correcting a specific mutation.

**FIGURE 1:**
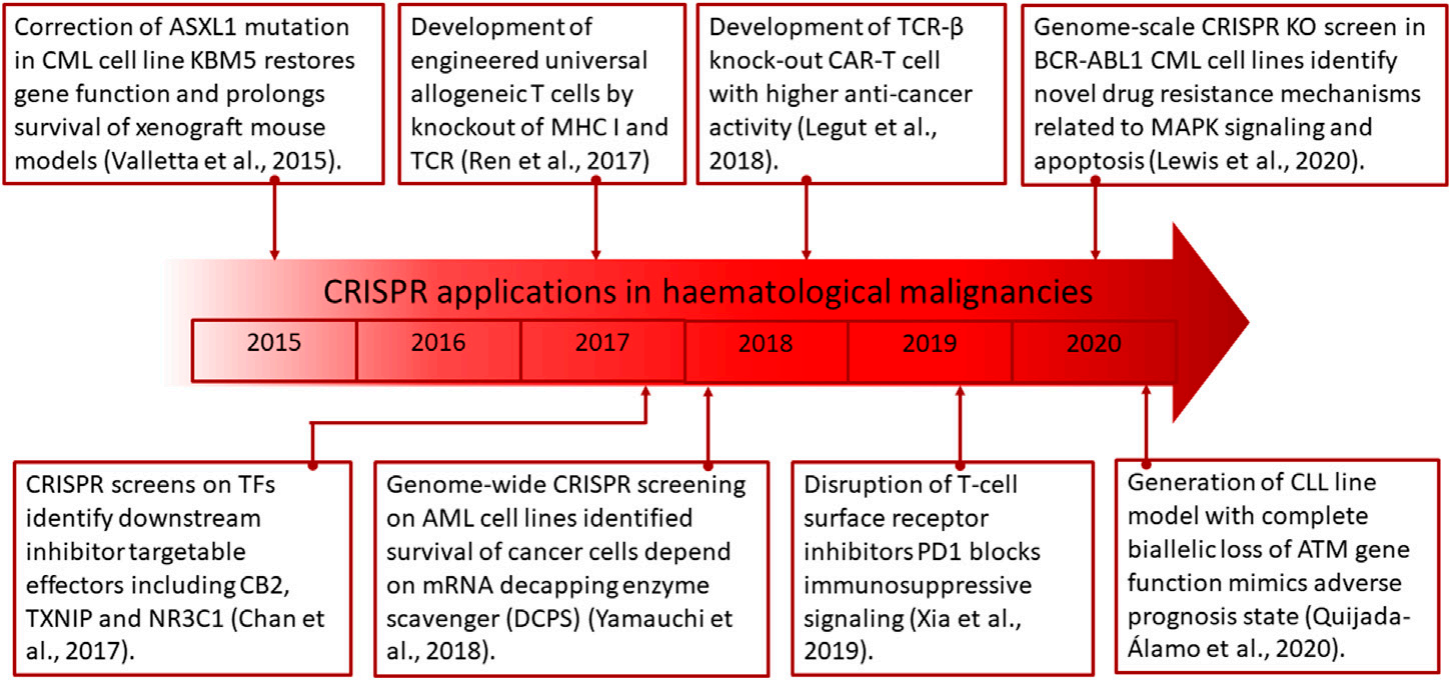
Timeline describing the research progress of CRISPR applications in hematological malignancies. Key studies contributing to major breakthroughs in the field are highlighted.

**FIGURE 2:**
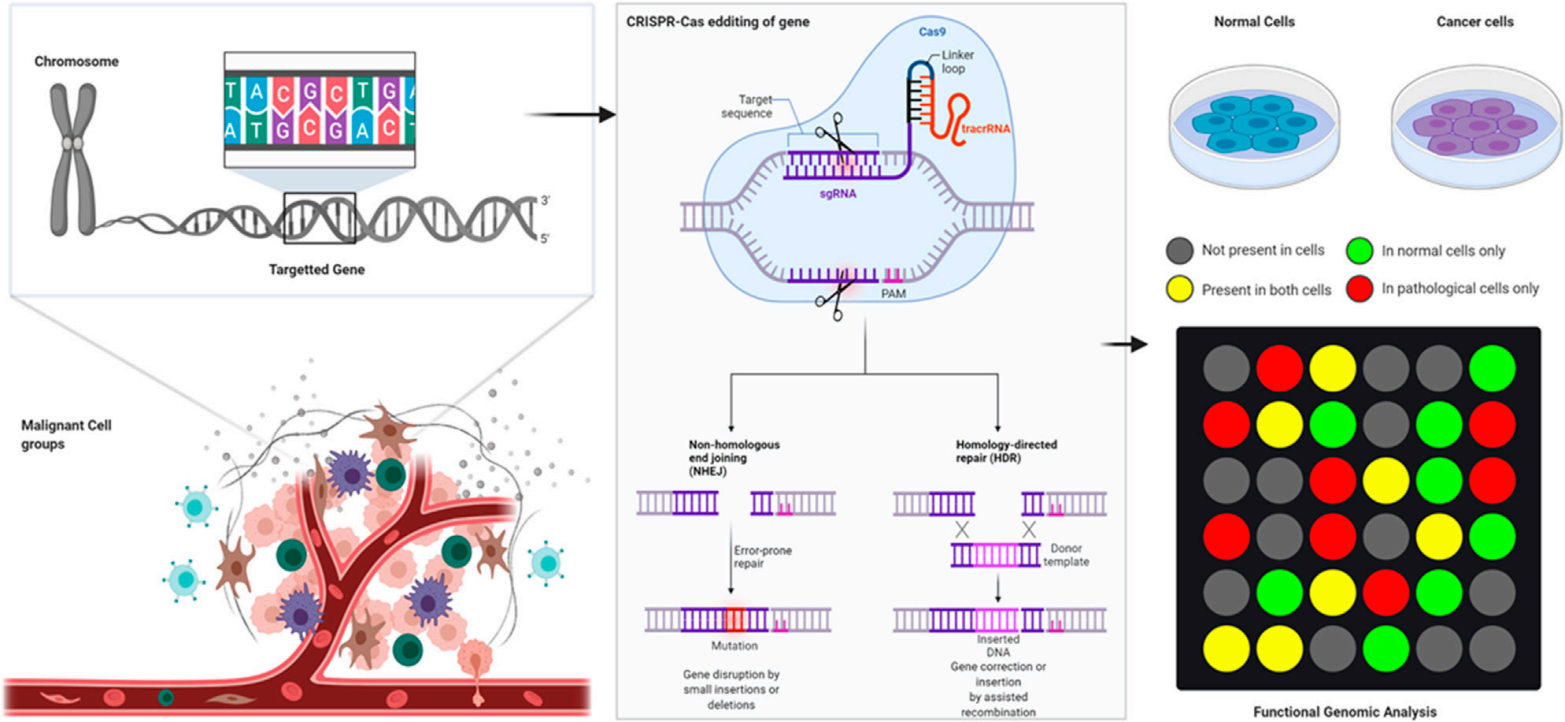
Functional Genomic analysis of hematological malignancies using CRISPR applications.

In conclusion, through current advancement in terms of gene editing, we can say that CRISPR has taken the research world by storm, evidently allowing us to make changes to the once impossible. CRISPR has made its way onto the freezers of labs all around the spectrum, easily accessible for researchers to use it. It is also becoming a mainstream methodology to study cancer biology given its versatility. It has now matured its way from experimental approach toward customized treatment involving cancer patients. CRISPR gene editing tools have also sparked significant advancements in enhancing our knowledge regarding hematological malignancies, which in essence presents us with potential therapeutic applications which holds much promise for alternative treatments for patients with this group of malignancies. Development of CRISPR utilization in studying the functional genomics of haematologic malignancies is depicted in Figure 1 and a general summary for studying functional genomics via CRISPR based tools is presented in Figure 2.
